# DOACs reduce risk for new-onset portal hypertension in patients with cirrhosis and atrial fibrillation: A retrospective cohort study

**DOI:** 10.1097/MD.0000000000047858

**Published:** 2026-03-13

**Authors:** Yunjuan Su, JingJing Wang, Qian Dong, Hao Guan, Changsheng Ma

**Affiliations:** a Department of Cardiology, National Center for Infectious Diseases, Beijing Ditan Hospital, Capital Medical University, Beijing, China; b Center of Liver Diseases, National Center for Infectious Diseases,Beijing Ditan Hospital, Capital Medical University, Beijing, China; c Department of Cardiology, Beijing Anzhen Hospital, Capital Medical University, Beijing, China.

**Keywords:** atrial fibrillation, cirrhosis, novel oral anticoagulants, portal hypertension

## Abstract

Patients with liver cirrhosis and atrial fibrillation (AF) pose a therapeutic challenge due to the conflicting risks of thrombosis and bleeding. The role of direct oral anticoagulants (DOACs) in this population remains uncertain. This study aims to evaluate the impact of anticoagulation therapy on new-onset portal hypertension and related complications in cirrhotic patients with AF. A retrospective cohort study was conducted in 502 hospitalized patients with cirrhosis and AF at Beijing Ditan Hospital (2010–2019). Clinical data, risk scores, and liver severity indices (Child-Pugh, Model for End-Stage Liver Disease) were collected. Propensity score matching was applied to minimize confounding. Primary endpoints were new-onset portal hypertension and all-cause mortality; secondary endpoints included gastrointestinal bleeding and thromboembolic events. Among 502 patients, 50 received oral anticoagulation. Anticoagulation was associated with a significantly lower risk of new-onset portal hypertension (*P* < .001) and gastrointestinal bleeding (*P* < .005) without increasing mortality (*P* < .003). After propensity score matching (n = 233), anticoagulation continued to reduce portal hypertension (*P* < .026) and gastrointestinal bleeding (*P* < .025), with no significant effect on mortality. Kaplan–Meier analysis confirmed improved survival in the anticoagulation group (*P* < .002). In cirrhotic patients with AF, anticoagulation, particularly direct oral anticoagulants, may lower the risk of portal hypertension and gastrointestinal bleeding without increasing mortality. Prospective studies are warranted to confirm these findings and guide clinical decision-making.

## 1. Introduction

This study aims to evaluate the impact of anticoagulation therapy on new-onset portal hypertension and related complications in cirrhotic patients with AF.The China Cardiovascular Health and Disease Report^[1]^ reveals a rising incidence of cardiovascular disease in China, with approximately 4.87 million individuals affected by atrial fibrillation (AF), making AF one of the most common arrhythmias in the nation. One of the AF complications is stroke, which, in severe cases can be incapacitating and leaves sequelae including hemiplegia of limbs.^[2]^ Oral anticoagulants are advised to prevent stroke and thrombosis in most patients with AF who show indication for anticoagulation. Although many real-world retrospective studies on the safety of anticoagulation therapy in special populations have been conducted, the balance between the risks and benefits of anticoagulation therapy in patients with cirrhosis and AF is still debatable and it has been thought that the risk of bleeding is considerably higher than that of normal patients due of coagulation dysfunction caused by decreased synthesis of coagulation factors by hepatocytes and decreased platelets in liver cirrhosis (LC).^[3]^ This complicates treatment choices since it is essential to reduce the risk of bleeding and prevent thrombosis at the same times.

Still, there is mounting evidence^[4,5]^ showing patients with cirrhosis have “rebalanced” or even hypercoagulable coagulation system. Anticoagulation therapy in patients with cirrhosis and portal hypertension is hypothesized to prevent liver decompensation, improve prognosis, and extend survival by reducing small thromboses in the minor portal vein branches and sinuses, so possibly preventing the progress of fibrosis. Rivaroxaban inhibits the active form of factor X, consequently reducing thrombin production. It also decreases portal hypertension in cirrhotic rats. However, the advantages and disadvantages of anticoagulation therapy for AF in Chinese patients with cirrhosis remain ambiguous. Clinical studies, such as Cerini F, focusing on the portal vein, indicate that enoxaparin can lower the incidence of portal vein thrombosis and decompensation events, while enhancing the survival rate of patients with advanced LC.^[6]^ Oral anticoagulants’ part in portal hypertension has not been well studied. While cirrhosis is connected to major changes in the coagulation cascade and pharmacokinetics, oral anticoagulants disturb the normal hemostatic process and increase the risk of hemorrhage. Those with chronic liver disease run more risk for thrombotic events as well as bleeding.

Therefore, this study investigated the effectiveness and safety of oral anticoagulants in patients with LC and AF hospitalized in Beijing Ditan Hospital by means of a retroactive study of patients. This study sought to evaluate for patients with AF and cirrhosis the safety, efficacy, and prognosis of oral anticoagulant drugs. To maximize the treatment impact and raise patient prognosis, it is expected to offer clinicians in the treatment decisions of patients with LC and AF stronger evidence and direction.

## 2. Method

### 2.1. Study population

This retrospective cohort included 502 hospitalized patients diagnosed with LC and AF at Beijing Ditan Hospital, Capital Medical University, from January 1, 2010 to December 31, 2019. This research adheres to the Declaration of Helsinki, sanctioned by the Ethics Committee of Ditan Hospital affiliated with Capital Medical University, and constitutes a retrospective study that does not require informed consent. We analyzed the incidence of endpoint events by gathering medical records during hospitalization, with primary endpoints comprising new-onset portal hypertension and all-cause mortality, and secondary endpoints including gastrointestinal bleeding, hemorrhagic stroke, and ischemic stroke. Hepatic encephalopathy, hepatorenal syndrome, ascites, portal hypertension, and portal vein thrombosis were recorded.

The diagnosis of LC is contingent upon the identification of endoscopic esophagogastric varices or ectopic varices in the gastrointestinal tract, in conjunction with epigastric ultrasound, transient elastography, or CT imaging that demonstrates features suggestive of LC or portal hypertension, such as splenomegaly, a portal vein diameter of ≥1.3 cm, and transient elastography results that fulfill the diagnostic criteria for different etiologies of LC. The diagnosis also comes from aberrant laboratory results including thrombocytopenia, leukopenia, hypoalbuminemia, hyperbilirubinemia, and prolonged prothrombin time. Portal hypertension-related complications including ascites, esophageal and gastric variceal hemorrhage, and hepatic encephalopathy cause compensated cirrhosis to be diagnosed as decompensated cirrhosis. The categorization of LC is determined by its etiology as follows: hepatitis virus infections (chronic hepatitis B, hepatitis C); alcoholic liver disease; nonalcoholic fatty liver disease (drug or chemical toxins); parasitic infections (schistosomiasis, clonorchiasis, etc); genetic and metabolic disorders (Wilson’s disease); circulatory disorders; autoimmune liver diseases (primary sclerosing cholangitis, autoimmune hepatitis); and cryptogenic cirrhosis.

The Child Grade is a predictive model that utilizes clinical (i.e., encephalopathy and ascites) and laboratory data to evaluate the severity of cirrhosis. The Model for End-Stage Liver Disease (MELD) scoring system comprises 5 parameters: serum bilirubin, creatinine (Scr), prothrombin time activity (INR), liver etiology, and serum sodium. The MELD score incorporates renal function and accounts for the critical complications of hepatorenal syndrome, particularly acute kidney injury, which are closely associated with the prognosis of patients with end-stage LC. This score offers a more accurate evaluation of LC severity and improves prognostic determination for patients with end-stage liver disease.

### 2.2. Data collection

Demographic information, clinical characteristics, and laboratory data were collected within 24 hours of admission. This encompassed age, sex, etiology of LC, presence of acute injury, complete blood count, liver function tests, prothrombin-international normalized ratio, Child-Turcotte-Pugh score, MELD score, serum sodium, and creatinine levels. The included covariates are: demographic characteristics; lifestyle factors and clinical status of comorbidities; medication history; and risk stratification scores for thromboembolism (CHA2DS2-VASc score) and bleeding (HAS-BLED score, which considers hypertension, renal/liver dysfunction, stroke, bleeding history or susceptibility, unstable international normalization ratio, older adults, and drug/alcohol use), MELD score, and Child score.

Data management: among the 503 included participants, baseline income information had 73 partially missing records (14.5%), and 21 partially missing records (4.2%) were observed at the 12-month follow-up. The missing data were determined to be missing at random. A single imputation method was applied: for normally distributed data, missing values were imputed using the mean, while for non-normally distributed data, the median was used for imputation. Data bias handling: to reduce selection bias, propensity score matching (PSM) was performed based on patient disease characteristics.

### 2.3. PSM analysis

The assignment of patients in this study to receive oral anticoagulation instead of randomization may introduce potential confounders and selection bias, thereby diminishing the reliability of the results. Thus, PSM is utilized to emulate random assignment and alleviate these effects. The logistic regression model was employed to calculate the propensity score using baseline characteristics such as INR, DBP, SBP, HR, WBC, PLT, B-type natriuretic peptide (BNP), ALB, LA, and EF. The caliper value is 0.2, and a 1-to-3 match is conducted. Two hundred eighty-three matched patients were used in subsequent analyses.

### 2.4. Statistical methods

Data are presented as mean ± standard deviation (SD), median (interquartile range, IQR), count, or percentage (%). The normality of the samples was evaluated utilizing SPSS statistical software. The Kolmogorov–Smirnov test and quantile–quantile plot were employed for analysis. For groups exhibiting non-normal distribution, continuous data were analyzed using either an independent Student’s *t* test or a Mann–Whitney *U*-test. Categorical variables were analyzed using the chi-square test or Fisher’s exact test. Univariate analysis was conducted using logistic regression to identify the risk factors associated with endpoint events. Odds ratios (ORs) and 95% confidence intervals (CIs) were computed. Additionally, we performed stratified analyses to assess whether the association between anticoagulation and mortality, as well as between anticoagulation and portal hypertension, differed across subgroups defined by sex, age, CHA2DS2-VASc score, HAS-BLED score, MLED, Child score, ejection fraction, systolic blood pressure (SBP), diastolic blood pressure (DBP), and heart rate (HR). Survival was assessed utilizing the Kaplan–Meier method, with *P* < .05 deemed statistically significant. SPSS (version 16.0; IBM, New York) or R software (version 3.3.2; R Development Core Team, Vienna, Austria). The sample size was estimated based on the primary outcome of portal vein recanalization rate. According to a prior study published in 2017,^[7]^ the recanalization rate was 71% in patients treated with anticoagulants therapy versus 42% in those patients that did not receive anticoagulants. Using the standard formula for comparing 2 independent proportions, with a 1-sided significance level (α) of 0.05 and a power (1 − β) of 80%, a minimum of 38 patients per group was required. Accounting for an anticipated dropout rate of 10% to 20%, we aimed to recruit at least 48 patients per group.


$$\begin{array}{l} \;n=\frac{2\bar{pq}{\left(z_{\alpha }+z_{\beta }\right)}^{2}}{{\left(p_{1}+p_{2}\right)}^{2}} \end{array}$$

## 3. Results

### 3.1. Patient characteristics

The study included 502 patients with cirrhosis and AF, consisting of 287 males (59.5%) with a mean age of 67.08 ± 11.48 years. The etiologies of LC included: hepatitis virus infections (chronic hepatitis B, hepatitis C) in 236 instances; 70 instances of alcoholic liver disease; 2 instances of nonalcoholic fatty liver disease (due to drug or chemical toxicity); 0 instances of parasitic infections (such as schistosomiasis, clonorchiasis); 0 instances of hereditary and metabolic disorders (e.g., Wilson’s disease); 26 instances of circulatory disorders; 72 instances of autoimmune liver diseases (including primary sclerosing cholangitis, primary biliary cholangitis, autoimmune hepatitis); a total of 96 instances of cryptogenic cirrhosis were recorded, with participants exhibiting 1, 2, or even 3 concurrent causes.

As of December 31, 2019, 139 out of 502 patients, representing 27.7%, succumbed to death. Fifty patients were administered oral anticoagulants and classified into 2 groups according to the prescription status recorded in medical records. The anticoagulant cohort was younger, displayed reduced heart rates, and showed markedly elevated levels of WBC, HGB, PLT, ALB, PT, Fib-4, and LA in comparison to the non-anticoagulant cohort. As of December 31, 2019, 139 out of 502 patients (27.7%) had died. Fifty patients were administered oral anticoagulants and classified into 2 groups according to the prescription status recorded in medical records. The anticoagulant cohort was younger, displayed reduced heart rates, and showed significantly elevated levels of WBC, HGB, PLT, ALB, PT, Fib-4, and LA in comparison to the non-anticoagulant cohort.

A subsequent comparison of baseline data between deceased patients and survivors indicated a reduced mortality rate in the anticoagulation group relative to the non-anticoagulation group. Moreover, the levels of PLT, HGB, ALB, and prothrombin time activity in the deceased cohort were markedly lower than those in the non-deceased cohort (*P* < .001). Conversely, age, creatinine level, WBC, BNP, aspartate aminotransferase , TBIL, DBIL, CRP, PT, FAB, FIB-4, aspartate aminotransferase to platelet ratio index, HAS-BLED, and MLED exhibited no significant differences. The child’s score in the death group surpassed that of the non-death group (*P* < .05). The levels of hemoglobin (HGB) and platelet (PLT) in the portal hypertension cohort were significantly lower than in the non-portal hypertension cohort (*P* < .001). Conversely, total bilirubin (TBIL), direct bilirubin (DBIL), and liver fibrosis indices were elevated compared to the non-portal hypertension cohort (*P* < .05). Additionally, coagulation indices were inferior in the portal hypertension cohort, apart from activated partial thromboplastin time (APTT) (*P* values: .014, <.001, .001, .001, respectively). The Child score portal hypertension group exhibited poorer outcomes compared to the group without portal hypertension (*P* = .003, <.001). No significant difference was observed between the CHA2DS2-VASC groups (*P* = .206), while the HASBLED score was elevated in the portal hypertension group (*P* < .001). A total of 240 cases of newly diagnosed portal hypertension were documented, along with 139 fatalities, 129 hospitalizations resulting from gastrointestinal hemorrhage, 70 ischemic stroke incidents, 55 occurrences of portal vein thrombosis, and 4 cases of intracerebral hemorrhage. The Child score portal hypertension cohort demonstrated inferior outcomes relative to the cohort without portal hypertension (*P* = .003, <.001). No notable difference was detected among the CHA2DS2-VASC groups (*P* = .206), whereas the HASBLED score was significantly higher in the portal hypertension group (*P* < .001). A total of 240 newly diagnosed cases of portal hypertension were recorded, alongside 139 fatalities, 129 hospitalizations due to gastrointestinal hemorrhage, 70 incidents of ischemic stroke, 55 occurrences of portal vein thrombosis, and 4 cases of intracerebral hemorrhage (Table 1) .

**Table 1:** The baseline of participants’ characteristics.

Variables	All Patients (n = 502)	Anticoagulation (n = 50).	Not anticoagulated (n = 452)	*P* values
Age (IQR, yr)	67.08 ± 11.48	65.56 ± 10.09	67.25 ± 11.62	.325
Male (%)	301 (60.0)	26 (52.0)	175 (38.7)	.096
HR (bpm)	83.37 (16.01)	79.38 (11.00)	83.81 (16.42)	.063
SBP (mm Hg)	117.83 (17.36)	120.72 (16.49)	117.51 (17.44)	.216
DBP (mm Hg)	71.32 (11.51)	70.22 (11.01)	71.44 (11.57)	.476
Habituation and comorbidities n (%)				
History of smoking	156 (31.1)	8 (16.0)	148 (32.7)	.023
History of alcohol consumption	166 (33.1)	9 (18.0)	157 (34.7)	.026
Hypertension	203 (40.4)	16 (32.0)	187 (41.4)	.259
Diabetes	161 (32.1)	19 (38.0)	142 (31.4)	.431
Coronary heart disease	138 (27.5)	9 (18.0)	129 (28.5)	.156
Complications n (%)				
Ascites	372 (74.1)	26 (52.0)	346 (76.5)	<.001
New-onset portal hypertension	240 (47.8)	146 (40.2)	94 (67.6)	<.001
Visceral embolism	10 (2.0)	4 (1.1)	6 (4.3)	.051
New gastrointestinal bleeding	129 (25.7)	45 (12.4)	84 (60.4)	<.001
Peripheral embolization	17 (3.4)	1 (2.0)	16 (3.5)	.874
New intracerebral hemorrhage	4 (0.8)	0 (0.0)	4 (0.9)	>.999
New cerebral infarction	70 (13.9)	7 (14.0)	63 (13.9)	>.999
Etiology of cirrhosis, n (%)				
(HBV\HCV)	236	29 (32.0)	207 (37.4)	<.001
Alcoholic liver disease	70	2 (4.0)	68 (15.0)	
Nonalcoholic fatty liver disease (drugs or chemical poisons)	2	0 (0.0)	2 (0.4)	
Parasitic infections (schistosomiasis, clonorchiasis, etc)	0	0 (0.0)	0 (0.0)	
Genetic, metabolic disorders (Wilson’s disease)	0	0 (0.0)	0 (0.0)	
Circulatory disorders	26	8 (16.0)	18 (4.0)	
Autoimmune liver disease (AIH\PSC\PBC)	72	2 (4.0)	70 (15.5)	
Cryptogenic cirrhosis	96	4 (8.0)	92 (20.4)	
CHA2DS2-VASc score	2.00 [1.00, 4.00]	2.00 [1.00, 4.00]	3.00 [1.25, 4.00]	.524
HASBLED	2.22 ± 0.90	2.00 ± 0.76	2.24 ± 0.91	.073
Liver function tests				
AST (IU/L)	36.00 [24.80, 64.35]	38.00 [24.75, 73.75]	35.90 [24.80, 62.20]	.522
ALT (IU/L)	20.60 [12.80, 41.90]	20.70 [13.43, 58.92]	20.60 [12.80, 38.40]	.425
GGT (IU/L)	53.30 [25.45, 92.00]	52.05 [25.55, 111.15]	53.60 [25.55, 92.00]	.505
Albumin (g/dL)	32.00 ± 5.83	35.28 ± 5.55	31.64 ± 5.75	<.001
TBIL (umol/L)	27.55 [15.90, 59.48]	16.95 [10.95, 31.05]	28.70 [17.15, 64.35]	<.001
Coagulation function				
APTT (s)	36.60 [31.50, 42.20]	38.30 [32.65, 43.70]	36.40 [31.50, 41.95]	.393
PTA (s)	63.00 [45.00, 78.00]	64.00 [48.00, 78.00]	45.50 [36.25, 70.75]	.002
Cardiac structure and function				
W (mm)	42.14 ± 8.29	49.08 ± 10.02	41.37 ± 7.71	<.001
LVED (mm)	46.70 ± 7.12	46.36 ± 8.49	46.73 ± 6.97	.725
LVSD (mm)	31.50 ± 6.67	32.40 ± 7.96	31.40 ± 6.51	.314
EF%	59.84 ± 9.56	57.96 ± 9.02	60.05 ± 9.60	.142
MELD score	11.40 [5.82, 17.93]	11.29 [5.74, 18.36]	12.30 [7.36, 15.84]	.917
Child score	8.00 [7.00, 10.00]	8.00 [7.00, 10.00]	7.00 [6.00, 8.75]	.021
Fibrosis scoring system				
Fib-4	7.21 [3.78, 13.89]	5.50 [3.58, 9.93]	7.40 [3.87, 14.38]	.055
APRI	1.33 [0.62, 3.04]	1.35 [0.64, 3.02]	1.02 [0.43, 3.62]	.413

AIH = autoimmune hepatitis, ALT = alanine aminotransferase, APRI = aspartate aminotransferase to platelet ratio index, APTT = activated partial thromboplastin time, AST = aspartate aminotransferase, DBP = diastolic blood pressure, EF% = ejection fraction, GGT = gamma-glutamyl transferase, HBV = hepatitis B virus, HCV = hepatitis C virus, HR = heart rate, IQR = interquartile range, LVED = left ventricular end-diastolic, LVSD = left ventricular systolic dysfunction, MELD = Model for End-Stage Liver Disease, PBC = primary biliary cholangitis, PSM = primary sclerosing cholangitis, PSM = propensity score matching, PTA = prothrombin time activity, SBP = systolic blood pressure, TBIL = total bilirubin.

### 3.2. Logistics regression analysis of anticoagulation therapy on the risk of new-onset portal hypertension events

Univariate logistic regression analysis was utilized to clarify the association between anticoagulation and endpoint events. Table 2 illustrates statistically significant differences in new-onset portal hypertension, mortality, and gastrointestinal bleeding between the 2 groups (OR [95% CI]. *P* > .310 [0.152–0.592], *P* < .001; 0.395 [0.159–0.847], *P* = .003; 0.185 [0.044–0.517], *P* = .005, Table 2). Anticoagulation diminishes the likelihood of new-onset portal hypertension, mortality, and gastrointestinal hemorrhage. After adjusting for age and sex, the odds ratio (OR) for the emergence of portal hypertension was calculated to be 0.295 (0.144–0.566), with a *P*-value of <.001, signifying statistical significance. Furthermore, the OR persisted below 1, recorded at 0.345 (0.164–0.680). Upon adjusting for variables such as gender, age, CHA2DS2-VASc score, HAS-BLED score, MELD score, and Child score (*P* = .003), and subsequently accounting for ejection fraction (EF%), systolic blood pressure (SBP), diastolic blood pressure (DBP), and heart rate (HR), the odds ratio for new portal hypertension events, which consistently remained below 1, retained statistical significance (all *P* < .005). Furthermore, anticoagulation demonstrated a correlation with a decrease in mortality (OR 0.401 [0.161–0.868], *P* = .031). However, no statistically significant variations were observed concerning sex, age, CHA2DS2-VASc score, HAS-BLED score, MELD score, Child score, ejection fraction, SBP, high blood pressure, and heart rate (*P* > .05, Table 3).

**Table 2:** Anticoagulation occurs with endpoint events logistics regression analysis.

	OR (95% CI)	*P*-value
New-onset portal hypertension	0.310 (0.152–0.592)	<.001
Death	0.395 (0.159–0.847)	.003
New-onset cerebral ischemia	1.005 (0.399–2.203)	.990
New intracerebral hemorrhage	0.003 (0.001–11.125)	.995
Peripheral embolization	0.556 (0.030–2.817)	.573
Visceral embolism	1.005 (0.054–5.513)	.997
New gastrointestinal bleeding	0.185 (0.044–0.517)	.005
Portal vein thrombosis	0.006 (0.002–1.292)	.986

CI = confidence interval, OR = odds ratio.

**Table 3:** Relationship of anticoagulation therapy to different endpoint events.

	Death		New-onset portal hypertension		New gastrointestinal bleeding	
	OR (95% CI)	*P*-value	OR (95% CI)	*P*-value	OR (95% CI)	*P*-value
Model 1	0.401 (0.161–0.868)	.031	0.295 (0.144–0.566)	<.001	0.168 (0.040–0.473)	.003
Model 2	0.464 (0.182–1.034)	.079	0.311 (0.149–0.609)	.001	0.147 (0.031–0.485)	.005
Model 3	0.550 (0.193–1.377)	.228	0.345 (0.164–0.680)	.003	0.180 (0.037–0.609)	.014
Model 4	0.534 (01.755–1.414)	.234	0.339 (0.160–0.676)	.003	0.172 (0.036–0.589)	.011

Model 1: Adjust gender, age.

Model 2: Adjust gender, age, CHA2DS2-VAScScore, HAS-BLED score.

Model 3: Adjust gender, age, CHA2DS2-VASc, HAS-BLED score, MELD score, and Child score.

Model 4: gender adjustment, ageCHA2DS2-VASc score, HAS-BLED score, MELD score, Child score, ejection fraction, SBP, HBP and heart rate.

CI = confidence interval, HBP = high blood pressure, MELD = Model for End-Stage Liver Disease, OR = odds ratio, SBP = systolic blood pressure.

### 3.3. Comparison of survival analysis between anticoagulant group and non-anticoagulation group

**Figure 1.**
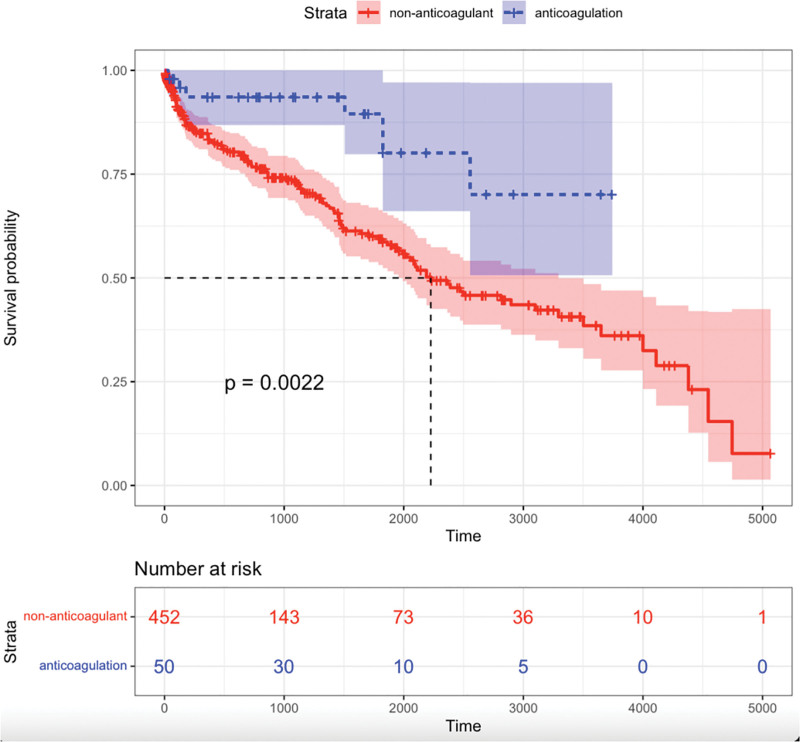
Kaplan–Meier analysis overall survival. The mortality rate was higher in patients without anticoagulation than patients with anticoagulation.

The Kaplan–Meier survival analysis demonstrated that the survival rate of the anticoagulation group significantly exceeded that of the control group (*P* = .002). The results underscore the beneficial influence of anticoagulation in enhancing the prognosis for patients with cirrhosis and AF, potentially informing clinical treatment strategies. (Fig. 1).

### 3.4. Subgroup analysis of risk factors for mortality

**Figure 2.**
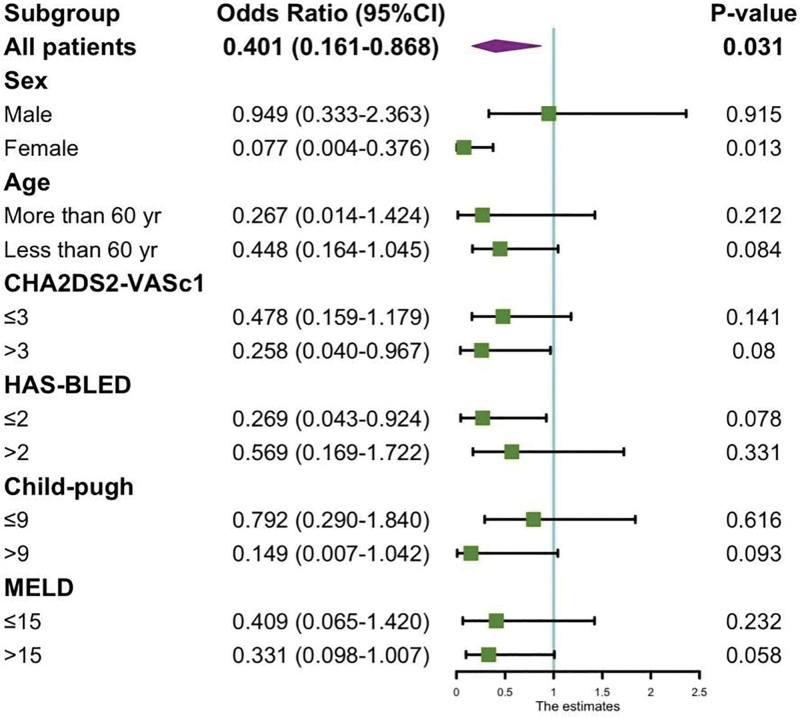
Subgroup analysis for the effect of anticoagulation in LC patients. Anticoagulation can be lowered in women with a C score of ≥3low risk of death (0.077 [0.004–0.376], *P* = .013). CI = confidence interval, LC = liver cirrhosis, MELD = Model for End-Stage Liver Disease.

The administration of anticoagulation has the potential to reduce the occurrence of new-onset portal hypertension and gastrointestinal bleeding within the general populace, especially among women aged over 60. Notably, the advantages are more pronounced in individuals presenting a CHA2DS2-VASc score of ≥3. The risk mortality was reduced in women (*P* < .05),0.077 (0.004–0.376), *P* = .013, and no statistical difference was found in other patients (*P* > .05, Fig. 2).

### 3.5. Subgroup analysis of anticoagulation therapy for new-onset portal hypertension events

**Figure 3.**
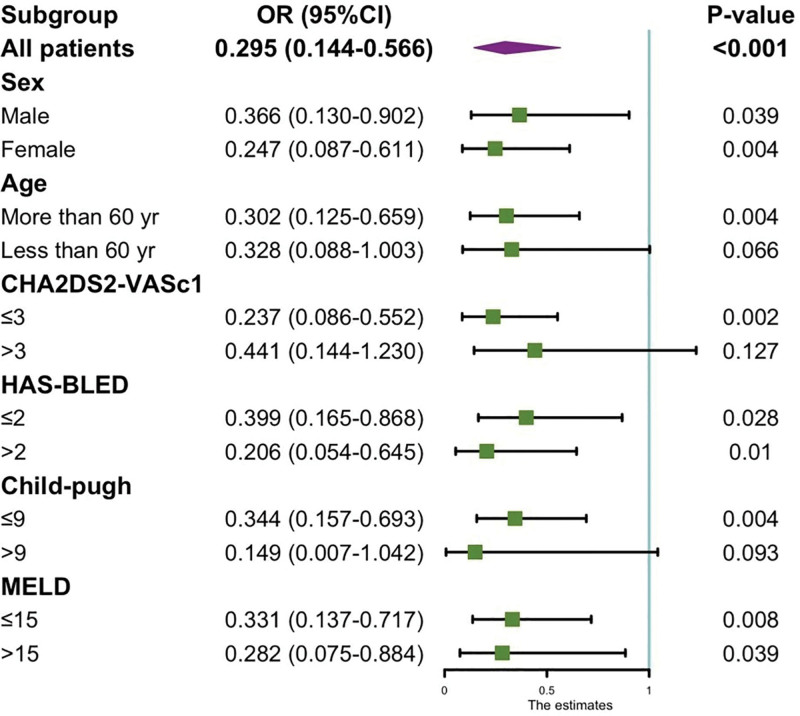
Subgroup analysis for the effect of anticoagulation in LC patients with Portal Hypertension. CI = confidence interval, LC = liver cirrhosis, MELD = Model for End-Stage Liver Disease.

The administration of anticoagulants could potentially reduce the likelihood of developing new-onset portal hypertension. Subgroup analysis revealed that women aged over 60 years experienced a more pronounced benefit from CHA2DS2-VASc ≥ 3, Child score < 9, and MELD score ≤ 15, while no statistically significant difference was noted in patients with CHA2DS2-VASc > 3 and Child score ≥ 10. *P* = .05, Figure 3.

### 3.6. Subgroup analysis of anticoagulation therapy for new-onset gastrointestinal events

**Figure 4.**
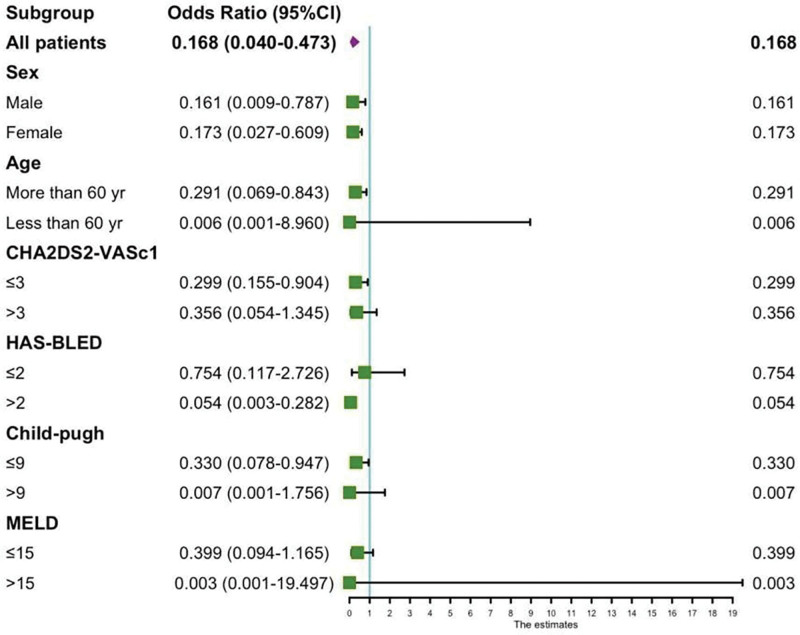
Subgroup analysis for the effect of anticoagulation in LC patients with bleeding events. Anticoagulation for women > 60 years of age, CHA2DS2-VASc ≤ 3, HAS-BLED3-5divide, Child5-9in subgroups of patients, Particularly protective against new gastrointestinal bleeding events *P* < .05). CI = confidence interval, LC = liver cirrhosis, MELD = Model for End-Stage Liver Disease.

Anticoagulation therapy can reduce the probability of subsequent gastrointestinal bleeding. Analysis of subgroups revealed that women aged over 60 experienced a more pronounced benefit, especially among those with CHA2DS2-VASc scores of ≤3 and Child scores ranging from 5 to 9. No statistically significant differences were noted among patients exhibiting CHA2DS2-VASc scores exceeding 3 and Child scores either equal to or >10 or <5 (*P* > .05, Fig. 4).

### 3.7. Effect of anticoagulation therapy on endpoint events after PSM

To address the confounding variables resulting from the disparity in a range of factors influencing prescriptions among anticoagulated and non-anticoagulated patients, we implemented a PSM approach with a 1:3 ratio. In the PSM analysis, we matched values for INR, DBP, SBP, HR, WBC, PLT, BNP, ALB, EF, as well as the CHA2DS2-VASc, HAS-BLED, Child, MELD, FIB-4, and APRI factors, resulting in a total of 233 patients from the entire cohort, of which 189 patients who did not receive oral anticoagulants were matched. The nearest neighbor algorithm demonstrated a commendable balance and even distribution of clinical features and laboratory parameters across these groups (all *P* > .5, Table 4). The logistic regression analysis of anticoagulation and endpoint events in the cohort post-PSM matching indicated that anticoagulation may decrease the incidence of new-onset portal hypertension and gastrointestinal bleeding, with odds ratios (95% CI) of 0.440 (0.206–0.885), *P* = .026, and 0.248 (0.058–0.728), *P* = .025, respectively. Moreover, the analysis revealed no statistically significant difference in mortality rates between the 2 groups following matching (*P* = .328, Table 5). Statistically significant differences were observed in the reduction of new-onset portal hypertension and gastrointestinal bleeding following additional adjustments for gender, age, CHA2DS2-VASc score, HAS-BLED score, MELD score, Child score, ejection fraction, SBP, high blood pressure, and heart rate (*P* < .05, Table 6).

**Table 4:** Characteristics in the PSM cohort.

Variables	All patients (n = 233)	Matched (complete) dataset	Not anticoagulated (n = 189)	*P* values
		anticoagulation (n = 44)		
Age (IQR, yr)	66.49 ± 11.83	65.48 ± 10.29	Select size 66.73 ± 12.18	.528
Male (%)	301 ± 60.0	24 ± 54.5	87 ± 46.0	.395
HR	80.94 ± 14.69	80.02 ± 11.12	81.16 ± 15.42	.645
SBP	120.52 ± 17.35	120.82 ± 17.45	120.44 ± 17.37	.898
DBP	70.42 ± 11.06)	70.59 ± 11.38	70.39 ± 11.02 a.m.	.912
BNP	290.40 [134.10, 624.80]	300.00 [152.67, 300.00]	273.70 [127.90, 772.00]	.243
AST (IU/L)	33.00 [22.70, 53.70]	36.80 [25.25, 75.73]	32.00 [22.30, 50.20]	.077
ALT (IU/L)	20.40 [12.50, 37.10]	19.55 [13.68, 61.87]	20.40 [12.30, 32.90]	.207
GGT (IU/L)	46.70 [23.90, 92.00]	52.05 [23.80, 94.42]	46.10 [23.90, 83.30]	.218
Albumin (g/dL)	33.74 ± 5.67)	34.96 ± 5.69	33.46 ± 5.64	.113
INR	1.61 ± 0.88	1.81 ± 1.13	1.56 ± 0.80	.085
CHA2DS2-VASc score	3.00 [1.00, 4.00]	2.50 [1.75, 4.00]	3.00 [1.00, 4.00]	.83
HASBLED	2.14 ± 0.86	Select size 1.93 ± 0.76	2.19 ± 0.87	.071
EF%	59.39 ± 9.15	58.27 ± 8.95	59.65 ± 9.20	.369
MELD score	9.40 [4.98, 14.09]	11.05 [5.80, 13.99]	8.98 [4.91, 14.09]	.33
Child score	7.00 [6.00, 9.00]	7.00 [6.00, 9.00]	7.00 [6.00, 8.00]	.999
Fibrosis scoring system				
Fib-4	5.57 [3.05, 10.18]	5.50 [3.60, 9.58]	5.59 [2.97, 10.40]	.804
APRI	1.01 [0.48, 1.99]	1.02 [0.47, 3.00]	1.01 [0.48, 1.86]	.418

ALT = alanine aminotransferase, APRI = aspartate aminotransferase to platelet ratio index, AST = aspartate aminotransferase, BNP = B-type natriuretic peptide, DBP = diastolic blood pressure, EF% = ejection fraction, GGT = gamma-glutamyl transferase, HR = heart rate, INR = prothrombin time activity, IQR = interquartile range, MELD = Model for End-Stage Liver Disease, PSM = propensity score matching, SBP = systolic blood pressure.

**Table 5:** Logistic regression analysis of anticoagulation and the occurrence of endpoint events.

	OR (95% CI)	*P*-value
Death	0.606 (0.198–1.532)	.328
New-onset portal hypertension	0.440 (0.206–0.885)	.026
New gastrointestinal bleeding	0.248 (0.058–0.728)	.025

CI = confidence interval, OR = odds ratio.

**Table 6:** The relationship between anticoagulation after PSM and new-onset portal hypertension and new-onset gastrointestinal bleeding events.

	New-onset portal hypertension		New gastrointestinal bleeding	
	OR (95% CI)	*P*-value	OR (95% CI)	*P*-value
Model 1	0.424 (0.198–0.858)	.021	0.239 (0.056–0.704)	.022
Model 2	0.466 (0.214–0.961)	.045	0.220 (0.044–0.767)	.032
Model 3	0.429 (0.193–0.902)	.030	0.229 (0.046–0.803)	.038
Model 4	0.436 (0.191–0.938)	.039	0.203 (0.040–0.751)	.030

Model 1: Adjust gender, age.

Model 2: Adjust gender, age, CHA2DS2-VASc1 rating, HAS-BLED score.

Model 3: Adjust gender, age, CHA2DS2-VASc score, HAS-BLED score, MELD score and Child score.

Model 4: Adjust gender, age, CHA2DS2-VASc, HAS-BLED score, MELD score, Child score, ejection fraction, SBP, HBP, and heart rate.

CI= confidence interval, HBP = high blood pressure, MELD = Model for End-Stage Liver Disease, OR = odds ratio, PSM = propensity score matching, SBP = systolic blood pressure.

## 4. Discussion

Events of stroke and systemic embolism stemming from AF often pose a serious threat to patients’ lives or markedly diminish their quality of life. Appropriate anticoagulation serves as a significant strategy to avert both new and recurrent strokes linked to AF. Events of stroke and systemic embolism arising from AF can often endanger patients’ lives or diminish their quality of life. Appropriate anticoagulation serves as a significant strategy to mitigate the risk of both new and recurrent strokes linked to AF. Children diagnosed with grade A cirrhosis exhibit robust compensatory liver function, yet they typically present no discernible clinical symptoms. In contrast, individuals with grade A cirrhosis or higher find themselves in a clinically decompensated state, characterized by marked liver dysfunction, portal hypertension, and frequently accompanied by gastrointestinal bleeding, ascites, secondary infections, hypersplenism, hepatic encephalopathy, carcinogenesis, and various multisystem complications. Consequently, the risk of mortality is markedly elevated in comparison to prior conditions. This empirical investigation involving patients diagnosed with AF and cirrhosis revealed a noteworthy decrease in the occurrence of new-onset portal hypertension and bleeding within the anticoagulation cohort when juxtaposed with the non-anticoagulation cohort, as evidenced by the CHA2DS2-VASc and HAS-BLED scoring systems, especially among female participants. These findings support the evidence presented in a comparable study carried out in Beijing, China. Furthermore, it offers substantial clinical evidence regarding the impact of oral anticoagulant therapy on portal hypertension in individuals suffering from AF and cirrhosis, while also laying a solid foundation for future anticoagulation treatments in patients with LC accompanied by portal hypertension.

This investigation presents a divergence from previous studies concerning individuals afflicted with AF and chronic cirrhosis. This research presents an empirical analysis of individuals diagnosed with AF and cirrhosis within the Beijing area of China. In Taiwan, earlier retrospective analyses of anticoagulation therapy among patients with cirrhosis and AF within Asian demographics indicated that such therapy correlated with a diminished risk of ischemic stroke and a favorable net clinical benefit when juxtaposed with no treatment in individuals suffering from AF alongside cirrhosis. Furthermore, the thromboembolic risk associated with direct oral anticoagulants (DOACs) was comparable to that of warfarin, while presenting a reduced incidence of major bleeding events. In practical application, medical professionals face difficulties in overseeing the entire range of coagulation (INR) in cirrhotic patients undergoing warfarin therapy, presenting a considerable risk that surpasses the intended parameters of the treatment. In individuals diagnosed with LC, the pharmacokinetic and pharmacodynamic characteristics of anticoagulants are modified, suggesting that lower dosages might be required to attain comparable anticoagulation effectiveness, thereby alleviating the deterioration of liver function. As a result, the consideration of thromboprophylaxis utilizing low-dose DOACs may be warranted for these patients.^[8-10]^ In 2019, Lee et al from Korea discovered that the efficacy and safety of anticoagulation therapy using DOACs surpassed that of warfarin in patients with AF and liver disease within the Korean population, a finding that also held true for individuals with notably active liver disease.^[10]^Secondly, to our knowledge, this study represents the inaugural comparison of portal hypertension in patients with AF and cirrhosis, thereby offering valuable evidence to inform anticoagulation therapy for individuals affected by cirrhosis and portal hypertension cirrhosis.^[11]^ Portal hypertension constitutes a notable complication in patients with cirrhosis, rendering its prevention and management essential for improving patient outcomes.^[12]^ Portal hypertension represents a significant complication in individuals afflicted with cirrhosis, making its prevention and management crucial for enhancing patient outcomes. Concurrently, intrahepatic microvascular thrombosis emerges as a significant pathological contributor to portal hypertension. The underlying mechanism may involve the elevation of portal vein pressure, which leads to the mechanical stretching of hepatic sinusoidal endothelial cells (LSECs). This stretching is detected by mechanoreceptor integrins present on their membranes, subsequently activating the Notch1 receptor. This activation fosters the secretion of the chemokine CXCL1, which serves to recruit neutrophils to the hepatic sinusoids, interacting with circulating platelets to precipitate thrombosis.^[13-15]^ Moreover, the interaction of thrombin with the protease-activated receptor family in hepatic sinusoidal cells facilitates the activation of hepatic stellate cells, induces vasoconstriction, and enhances collagen synthesis. Additionally, it encourages LSECs to recruit and adhere to leukocytes, thereby promoting thrombosis. Portal hypertension constitutes a notable complication in patients with cirrhosis, rendering its prevention and management essential for improving patient outcomes. Research involving animal models has demonstrated that anticoagulant medications, including natreparin and enoxaparin, can mitigate liver fibrosis and portal hypertension in rats.^[16,17]^ Rivaroxaban acts by inhibiting the active form of factor X, which in turn suppresses thrombin production, leading to a reduction in portal hypertension in rat models of cirrhosis.^[18]^ Nonetheless, the number of clinical trials investigating oral anticoagulants to mitigate portal hypertension remains limited. Current clinical studies indicate that enoxaparin has the potential to decrease the occurrence of portal vein thrombosis and decompensation events, thereby enhancing the survival rate of patients suffering from advanced cirrhosis.^[19]^ A recent investigation revealed that in individuals suffering from portal hypertension, the administration of anticoagulants led to enhanced outcomes and extended survival, irrespective of portal vein recanalization, while the precise mechanisms underlying this phenomenon remain unexplored.^[20]^ Furthermore, certain researchers have suggested that the intima of the portal vein wall exhibits thickening in individuals afflicted with LC, whereas it is notably absent in healthy individuals.^[21]^ In individuals diagnosed with portal vein thrombosis, there is a progression of portal intimal thickening, often accompanied by intraluminal fibrin-rich thrombosis. Most cases of portal vein thrombosis are characterized by a thickened intima of the portal vein wall. Researchers have suggested that portal vein occlusion or stenosis may represent a more precise mechanism than what is currently classified as cirrhosis-related portal vein thrombosis. Moreover, a mild degree of portal endartial hyperplasia has been noted in individuals with cirrhosis in the absence of portal vein thrombosis.^[22]^

Furthermore, anticoagulant therapy spans multiple disciplines and conditions, and an analysis of case studies featuring clinical pharmacists in anticoagulation consultation and active monitoring illustrates the collaboration among physicians, pharmacists, and laboratory specialists to guarantee the safe and effective administration of anticoagulant medications.^[23]^ The physiological characteristics of Asian populations have heightened clinicians’ apprehensions regarding the prescription of anticoagulants, particularly the administration of low-dose DOACs, owing to the associated bleeding risks. Notwithstanding this, our study revealed a clinical advantage for patients administered oral anticoagulants. Our investigation is grounded in empirical data that more accurately represents prescribing behaviors and standard clinical practices in China. Previous comprehensive community-based comparative studies of DOACs and warfarin in the context of chronic liver disease with AF have solely encompassed individuals exhibiting normal liver function, thereby excluding participants with active liver disease or decompensated cirrhosis from the study cohort. This study included 502 hospitalized patients diagnosed with cirrhosis complicated by AF. Of these, only 50 patients (9.1%) were administered oral anticoagulation therapy, while a substantial majority, 452 patients (90.9%), did not receive such treatment. This lack of anticoagulation therapy notably heightened the risk of subsequent thrombosis and complications associated with portal hypertension.

The foremost advantage of our research lies in the inclusion of all qualifying patients from Beijing Ditan Hospital, ensuring an exhaustive follow-up process. As a result, the probability of selection bias was negligible. Moreover, our patients demonstrate a meticulous evaluation of liver function, which is frequently elusive in numerous retrospective database studies. Our investigation is subject to various constraints. Despite being the most extensive study of cirrhosis with AF conducted in Beijing, China, a mere 10% of patients were administered anticoagulation, thereby diminishing the statistical precision of our estimates. We did not evaluate our exposure as a changing over time variable. Patients without cirrhosis could acquire cirrhosis during the study; hence, the inability to monitor these changes could result in misclassification bias. This distortion of inequalities might lead to an underestimating of results. One also runs the danger of uncontrollably mixing. For instance, oral anticoagulants may be prescribed to patients with the lowest clinical risk of bleeding; hence, this selective treatment could cause an underestimating of the risk of bleeding complications in users of oral anticoagulants. Medical records are not recorded, and ischemic stroke in patients with cirrhosis is not defined as thrombotic or embolic stroke. One cannot totally exclude the effects of these confounders.

This research produced noteworthy findings regarding the systematic evaluation and effectiveness analysis of oral anticoagulants in patients with cirrhosis and AF. Initially, regarding efficacy, patients prescribed oral anticoagulants have demonstrated notable benefits in the prevention of thromboembolic events. This indicates that suitable anticoagulation can be both practical and beneficial for individuals suffering from cirrhosis and AF. Furthermore, there may be discrepancies in both effectiveness and safety among various categories of oral anticoagulants. Future research must delve deeper into the application characteristics of specific anticoagulant medications within this unique population, thereby equipping clinicians with more precise guidance for treatment. Moreover, the extent of cirrhosis could influence the effectiveness and safety of oral anticoagulants. This study stratified the extent of cirrhosis; however, larger sample sizes and more rigorously stratified investigations are necessary to clarify this association. Furthermore, the effectiveness of the treatment, which may require the use of additional medications, could impact the therapy’s overall efficacy, thus demanding careful deliberation in clinical practice. The study presents certain limitations, notably a small sample size and a limited number of patients on oral anticoagulants. Additionally, the follow-up duration may not suffice to comprehensively assess long-term efficacy and adverse events. Furthermore, the influence on all-cause mortality could be compromised by the constraints of follow-up events and sample size. Recent observational studies suggest that DOACs demonstrate remarkable safety and efficacy in patients with cirrhosis; nonetheless, further research with larger sample sizes is essential to validate these conclusions.^[24]^ Furthermore, given the significant individual variability among patients with cirrhosis, there may exist certain confounding factors that have not been thoroughly addressed. Future investigations ought to broaden sample sizes, prolong follow-up durations, and implement more stringent study designs to mitigate bias.

## 5. Summary

In summary, the use of oral anticoagulant therapy in patients with cirrhosis and AF has the potential to reduce the risk of overall mortality and the occurrence of portal hypertension, indicating a degree of efficacy alongside certain risks. In developing a treatment plan for these patients, it is imperative for clinicians to conduct a comprehensive evaluation of the patient’s liver function, thromboembolic risk, and bleeding risk. They must carefully weigh the advantages and disadvantages, tailor the choice of suitable anticoagulant medications and dosages to the individual and enhance monitoring protocols to guarantee the safe and effective management of patients. In parallel, there is a pressing need for additional high-quality studies and improved prospective research data in the future to refine treatment options within this field.

## Acknowledgments

The authors thank all the staff in this study for their dedication, support and hard work.

## Author contributions

**Conceptualization:** Yunjuan Su, Qian Dong, Changsheng Ma.

**Data curation:** Yunjuan Su, JingJing Wang, Qian Dong, Hao Guan, Changsheng Ma.

**Formal analysis:** Changsheng Ma.

**Investigation:** Yunjuan Su, JingJing Wang.

**Methodology:** Hao Guan, Changsheng Ma.

**Project administration:** Changsheng Ma.

**Resources:** Yunjuan Su, JingJing Wang, Qian Dong.

**Supervision:** Yunjuan Su, Hao Guan.

**Validation:** Yunjuan Su, Qian Dong.

**Visualization:** Yunjuan Su, JingJing Wang, Qian Dong, Hao Guan, Changsheng Ma.

**Writing – original draft:** Yunjuan Su, JingJing Wang, Changsheng Ma.

**Writing – review & editing:** Yunjuan Su, Qian Dong, Hao Guan.
